# The Heterogeneous Impact of Internet Use on Older People’s Mental Health: An Instrumental Variable Quantile Regression Analysis

**DOI:** 10.3389/ijph.2023.1605664

**Published:** 2023-03-07

**Authors:** Huoyun Zhu, Zhaoqi Li, Wenyi Lin

**Affiliations:** School of Public Administration School of Emergency Management, Institute of Common Prosperity and National Governance, Jinan University, Guangzhou, China

**Keywords:** mental health, internet use, heterogeneity, older people, IV quantile regression

## Abstract

**Objectives:** Whether Internet use improves older people’s health is an open question. This study empirically investigated the impact of Internet use on older people’s mental health with a focus on the heterogeneity among subgroups.

**Method:** Data come from the 2018 China Health Retirement Longitudinal Study (*n* = 8,505). An instrumental variable quantile regression method (IVQR) combines the instrumental variable and quantile regression to resolve the endogeneity and heterogeneity generally challenged in ordinary least squares (OLS).

**Results:** Although Internet use generally improves older people’s mental health, there is enormous heterogeneity in the effects on older adults with different mental health conditions. Specifically, Internet use only has a mitigating impact on older adults with poor mental health. Those heterogeneities are also found between rural and urban residents but not between genders.

**Conclusion:** Our findings shed light on active and healthy aging strategies. Two policy priorities include, on the one hand, the Internet user environment should be improved in parallel with Internet technology; on the other hand, multiple measurements are urgent to be developed to deal with the heterogeneity and unevenness of the impact of Internet technology on older people.

## Introduction

Aging combined with digitalization is the most prominent feature in the Post-epidemic era. Population aging in China is accelerating. The Seventh Population Census shows that in China, older people aged 65 and above reached 190 million in 2020, accounting for 13.5 percent of the total population (1). It is expected to be 398 million by 2060 (2). On the one hand, older people’s physical functions decline with age; on the other hand, due to the increasing “miniaturization” of the traditional family structure and withdrawal from society and the labor market, older people are vulnerable to health risks. Compared to physical health, mental health is often overlooked. Older people’s mental health in China is not optimistic (3). More than 85 percent of older people suffer from varying psychological problems, and 27 percent are in a severe phase (4). Appropriate intervention measures for older people’s mental health reduce medical care expenditure but also help to alleviate the conflict between the supply and demand of social services to improve their quality of life.

In the post-epidemic era, the digitalization of prevention measures has made the Internet gradually become an essential part of older people’s life. It also results in an increasing number of older cyber citizens. The size of older cyber citizens aged 60 and above in China has increased from 40 million in 2017 to 119 million by the end of 2021, the proportion of older cyber citizens to older people aged 60 and above has accordingly increased from 16.6 percent to 43.2 percent (5). Meanwhile, the Fifth Plenary Session of the 19th CPC Central Committee put forward the comprehensive promotion of a healthy China, in which the health aging route has been outlined. This report also emphasizes the academic research and application between the Internet and healthy aging at the social level. It was followed by a series of policies on “smart aging” and “Internet + Aging.” Therefore, it is of great theoretical and practical value to investigate the impact of Internet use on older adults’ mental health and the heterogeneity among subgroups.

Many scholars have discussed the relationship between Internet use and older people’s mental health with distinct conclusions. Generally, there are three different attitudes to the relationship between Internet use and older people’s mental health. The first view supports the positive effect of Internet use on older people’s mental health. Boekel (6) pointed out that Internet use allows older people to expand or maintain social connections, boosts access to information, and thus reduces feelings of isolation. Cotten et al. (7) found that older people who use the Internet suffer from less depression and enjoy better mental health. Some studies in China have also found that Internet use can promote older people’s mental health through mediating pathways such as social interaction (8–10).

The second opinion, however, supports the adverse effect. Some studies proposed the “Internet social paradox”, which states that Internet users are prone to loneliness (11, 12). Because older cyber citizens reduce social contact and participation, negatively affecting their mental health (13). Meanwhile, the increased frequency of Internet use may lead to problems such as sedentary lifestyles and obesity (14). Wan et al. (15) interviewed 363 older adults in Community Day Care Centres in Henan Province. They concluded that older adults who used their mobile phones for longer average daily hours or used an excessive number of software functions are likely to be higher levels of loneliness. It suggests that a battery of older people are “Silver-haired phubbers” who spend much time on mobile games, short videos, and chatting (16). Over-dependence on the Internet and inappropriate use of its functions generate older people’s mental and social adjustment disorders (17).

The third one supports neither above points. The Internet does not provide emotional support for users if online relationships do not translate into offline interactions (18). Chen et al. (19) argued that the positive effects of Internet use on mental health are diminishing, and older people on the Internet may also experience the dilemma of “being alone together.” Hong et al. (20) found that Internet use led to higher social connectivity, greater empathy, and social cognition but did not affect their sense of isolation in interviews with 54 older people.

Due to the conflicting views, the heterogeneity of the impact of Internet use on older people’s mental health has gradually been brought to the attention of scholars. However, conclusions on the impact of heterogeneity among subgroups are substantially varied. Some studies found significant urban-rural differences in the impact of Internet use on older people’s physiological and mental health. Urban older people benefit more than their rural counterparts (21, 22). Li and Zhou (23) examined the moderating effect of gender on the mediatory role of parent-child connectedness between Internet use and subjective wellbeing. In contrast, Li and Jia (24) found that Internet use had a more substantial effect on mental health promotion for the oldest old, male, and rural older people. Another study found that the effect of Internet use on health promotion diminished with age (25).

This literature review reveals that there is no consensus on the influence of Internet use on older people’s mental health, and there are some limitations. First, most studies treat older people as a homogeneous group and conclude varied, even conflicting outcomes. A tiny amount of research on heterogeneity mainly focuses on genders, geographic areas, and other variables. Scarce studies explore the uneven impact of Internet use on subgroups with different mental health conditions. Second, in terms of research methodology, existing studies mainly focused on simple descriptive statistics and multiple linear regression analysis, which have been well criticized for the estimation effectiveness, especially in terms of endogeneity and heterogeneity. For example, Internet use and older people’s mental health may interact.

This study at hand fills those gaps and is divided into three sections. First, the dataset, variable measurements, and method used in this study are described. Second, the empirical results are reported. The final section of this study discusses the empirical results followed by the policy suggestions for future direction to improve older people’s mental health and reduce inequality in health.

## Methods

### Data

This study employs data from the China Health Retirement Longitudinal Study (CHARLS), a nationally representative survey initiated in 2011, followed by three follow-up waves in 2013, 2015, and 2018, respectively. It has high national representativeness (9). The response rate and data quality are among the highest in the world for similar projects (8). This survey has covered 28 provinces, 150 county-level units, and 450 village-level units. Considering the availability of Internet use information only in the 2015 and 2018 waves and the higher Internet coverage rate in the latter one, the dataset adopted in this study is from the last wave in 2018, with 19,816 middle-aged and older people, and the Internet use information from the 2015 wave was used as an instrumental variable. This study confines a sample between 60 and 80 years old, given their higher health status rates and Internet use rates. The sample size reduces to 9,176, which further reduces to 8,505 after eliminating missing data.

### Variable Measurement

#### Dependent Variable: Depression Symptoms

This study uses the CES-D scale to measure older people’s mental health, which is the most widely used tool for measuring depressive symptoms. This scale has demonstrated high levels of internal consistency across samples and concurrent validity (26), and has been validated for use in Chinese contexts (27). The Cronbach’s alpha based on the used Chinese sample is 0.809, suggesting high reliability. The CES-D scale includes ten items covering both negative and positive aspects. These are: *“I was bothered by things that do not usually bother me,” “I had trouble keeping my mind on what I was doing,” “I felt depressed,” “I felt everything I did was an effort,” “I felt hopeful about the future,” “I felt fearful,” “My sleep was restless,” “I was happy,” “I felt lonely,” “I could not get going*.*”* As for the negative questions, the answer values were coded as follows: rarely or none of the time (<1 day) = 0; some or a small amount of the time (1–2 days) = 1; occasionally or a moderate amount of the time (3–4 days) = 2; most or all of the time (5–7 days) = 3. It was coded in a reverse direction for the positive questions. We then made a sum of ten items—the scores of mental health range from 0 to 30. A higher score indicates more significant depression (28).

#### Independent Variable: Internet Use

The independent variable in this study is Internet use, which was measured by the frequency of Internet use among older people (12). It is measured by two questions, including “*Have you done any of these activities in the last month?*” and “*How often in the last month did you do them?*” The first question covers 11 activity patterns, such as Internet use, charity work, and social contacts, companied by an additional option indicating without any activities. Respondents were asked for all activities one by one. Then the second question was employed to measure the participation frequency of each activity with three degrees: not regularly, almost every week, and almost daily. We selected the Internet use activity in the first question and calculated the Internet use frequency variable based on the second one, which was assigned 1, 2, 3, and 4 to “No,” “Not regularly,” “Almost every week,” and “Almost daily,” respectively.

#### Covariates: Demographic Characteristics

We control for a range of covariates including individual and household characteristics that may be correlated with Internet use and older people’s mental health (9). In light of previous studies, we selected gender (male = 1; female = 0), age, ethnicity (Han = 1; Minority = 0), educational attainment (illiteracy = 1; primary school = 2; middle school = 3; high school and above = 4), religiosity (yes = 1; no = 0), residence status (urban areas = 1; rural areas = 0), marital status (married = 1; others = 0), and coresidence with children (yes = 1; no = 0) (29, 30).

#### Instrumental Variable: Internet Use in 2015

Given a potential reverse causal relationship between Internet use and older people’s mental health, we have to consider the endogeneity issue, which may result in estimation bias. A majority of scholars used propensity score matching to address it, relying on the “observable factors are ignored” hypothesis. In contrast, the instrumental variable method has unique advantages for quantitative analysis based on survey data (31). This study selected the one-period lagged Internet use as the instrumental variable-the Internet use variable in the 2015 wave. Older people’s Internet use in the 2015 wave is highly associated with their Internet use in the next wave, while it is not correlated with the disturbance term (9).

Table 1 reports the variables and sample distribution. It shows that the average depression scores are 8.8 indicating an upper level of health conditions. More older people had access to the Internet in 2018 than in 2015, although most of them did not use the Internet regularly. Another point of concern is that 54.3 percent of older adults live apart from their children, who are at higher risk of experiencing mental health problems.

**TABLE 1:** Sample distribution (N = 8,505) (China Health and Retirement Longitudinal Study, China, 2015, 2018).

Variables	Minimum value	Maximum value	Average value	Standard deviation
Depression Scores	0	30	8.762	6.646
Internet use (2018)	1	4	1.178	0.695
Internet use (2015)	1	4	1.084	0.482
Gender	0	1	0.496	0.500
Age	60	80	67.705	5.341
Ethnicity	0	1	0.929	0.258
Education attainment	1	4	2.079	0.906
Religiosity	0	1	0.213	0.409
Marital status	0	1	0.822	0.382
Coresidence with children	0	1	0.543	0.347
Residence	0	1	0.248	0.432

### Empirical Strategy

We first used ordinary least squares (OLS) and two-stage least squares (2SLS) regression to estimate the following models to explore the effect of Internet use on older people’s depression scores.

$${Health}_{i}=\alpha {Internet}_{i}+\gamma X_{i}+\mu_{i}$$ (1)

$${Internet}_{i}=\gamma_{1}X_{i}+\gamma_{2}Z_{i}+\varepsilon_{i}$$ (2a)

$${Health}_{i}=\alpha_{1}X_{i}+\alpha_{2}{Internet}_{i}+e_{i}$$ (2b)

*Health*_*i*_ is the mental health status (depression scores) of individual *i*. *Internet*_*i*_ is the Internet use frequency of individual *i*, and *X*_*i*_ represents all covariates, including gender, age, ethnicity, marital status, etc. *Z*_*i*_ is the instrumental variable related to *Internet*_*i*_, and *μ*_*i*_ is the error term.

The OLS and 2SLS deal with the average treatment effect of the independent variable on the dependent one, which cannot resolve the heterogeneity among subgroups. We then employed the quantile regression method with instrumental variables (IVQR) proposed by Chernozhukov and Hansen (32) to compare the heterogeneity among subgroups with different depression scores. We estimated the *τ* ^*th*^ quantile of the dependent variable, Health, as a linear function of the endogenous metric (*Internet*_*i*_), a vector of covariates (*X*_*i*_), and a disturbance term (*μ*_*i*_).

$$Q_{health}\left(\tau \right)=\alpha_{\tau }{Internet}_{i}+\beta_{i}X_{i}+\mu_{i}$$ (3)

We further assume ${Internet}_{i}$ to be predicted by the following equation based on Eq. 2a.

$${Internet}_{i}=\gamma_{1\tau }X_{i}+\gamma_{2\tau }Z_{i}+v_{i}$$ (4)

Where *v*_*i*_ denotes a disturbance term being similar to $\varepsilon_{i}$ in Eq. 2a. The quantile regression model at the *τ* ^*th*^ quantile of *Health*_*i*_ is then identified by:

$$Ρ\left[{Health}_{i}\leq \alpha_{\tau }{Internet}_{i}+\beta_{\tau }X_{i}+\mu_{i}|X_{i},Z_{i}\right]=\tau$$ (5)

This leads to the simplified objective function:

$$\arg\min\overset{n}{\underset{i=1}{\sum }}\rho_{\tau }\left({Health}_{i}-\alpha_{\tau }{Internet}_{i}-\beta_{\tau }X_{i}-\gamma_{\tau }Z_{i}\right)$$ (6)

Where *ρ*_*τ*_(*) denotes the quantile loss function, and then, we could estimate the coefficient ατ by tackling the minimization problem.

We conducted all empirical analyses by Stata version 16.0.

## Results

### The Impact of Internet Use on Older People’s Mental Health

We begin with a multiple linear regression model to analyze the effect of Internet use on depression scores in older people, as shown in Table 2. Model 1 only contains Internet use. The coefficient of Internet use is −1.215 with a statistical significance at 0.1 percent level, suggesting a positive health promotion effect. Model 2 adds the individual characteristics variables based on Model 1. The Internet use coefficient remains robust but significantly reduced to −0.581. Model 3 further introduces family characteristics variables, the results of which are consistent with the above two models. We can preliminarily conclude that the higher the frequency of Internet use, the lower the depression score and the better the mental health status of older people.

**TABLE 2:** The effect of Internet use on depression scores of older people (China Health and Retirement Longitudinal Study, China, 2018).

Variable name	Model 1	Model 2	Model 3
Internet use	−1.215*** (0.103)	−0.581*** (0.108)	−0.574*** (0.108)
Gender (female = 0)		−1.809*** (0.152)	−1.653*** (0.154)
Age		0.011 (0.013)	−0.010 (0.014)
Ethnicity (other ethnic groups = 0)		−0.558** (0.271)	−0.496* (0.271)
Educational attainment (illiterate = 1)			
Primary Schools		−0.465*** (0.177)	−0.450** (0.177)
Junior High School		−1.488*** (0.240)	−1.455*** (0.239)
High School and above		−2.085*** (0.297)	−2.040*** (0.297)
Religiosity (others = 0)		−0.682*** (0.175)	−0.694*** (0.175)
Residence (rural = 0)		−1.258*** (0.176)	−1.257*** (0.176)
Marital status (others = 0)			−1.272*** (0.190)
Coresidence with children (no = 0)			−0.156 (0.140)
Constant term	10.193*** (0.141)	11.253*** (0.955)	13.615*** (1.023)
Observations	8,505	8,505	8,505
R^2^	0.016	0.066	0.071

Note: ***, **, * denotes 1%, 5% and 10% significance levels, respectively; all coefficients and S.E. are unstandardized.

As for control variables, gender, education, marital status, residence, and religiosity significantly affect older people’s mental health except for age and coresidence with their children. The regression coefficient of gender is significantly negative, indicating that women’s health status is worse than men’s. While educational attainment is positively related to mental health, suggesting better health status for those with higher education. Both religion and residence status (urban or rural) are negatively associated with older people’s health. Those living in urban areas and having religious beliefs show lower depression scores, which is consistent with the findings of Li (33). Among the family characteristics variables, marital status significantly negatively affected older people’s mental health. Those with a spouse are in better health (21). Interestingly, older people’s mental health does not worsen with age and is not associated with whether they reside with their children.

We then conducted the 2SLS with the first-order lagged Internet use as the IV to test the potential endogeneity. The validity of the selected instrumental variables was tested first, and the results showed that both the Wald-F statistic and the KP Wald-F statistic were much more significant than 10, indicating a refusal of a weak instrumental variable. Secondly, there is no over-identification problem because the number of endogenous variables is consistent with the number of instrumental variables.

The detailed results of the 2SLS analysis are reported in Table 3, which confirms previous results based on the OLS. Moreover, the coefficient of Internet use in the second stage is −0.854, more significant than in OLS. It indicates an underestimation by a traditional linear regression and confirmation of a robust causal relationship between Internet use and mental health in old age.

**TABLE 3:** Regression of 2SLS model for the effect of Internet use on depression scores of older people (China Health and Retirement Longitudinal Study, China, 2018).

Variable name	Stage 1	Stage 2
Internet use (2015)	0.549*** (0.032)	
Internet use (2018)		−0.854*** (0.209)
Control variables	Yes	Yes
Constant term	0.946*** (0.094)	14.052*** (1.080)
Observations	8,505	8,505

Note: ***, **, * denote 1%, 5%, and 10% significance levels, respectively; all coefficients and S.E. are unstandardized.

### Heterogeneity of Health Promotion Effects

We then explore the heterogeneous impact of Internet use on older people’s mental health using IVQR. As shown in Table 4, Internet use coefficients vary among subgroups and decrease steadily from −0.064 for the first quantile with the lowest depression scores to −2.220 for the ninth quantile. This confirms that traditional OLS regression treats all older people equally and masks the considerable heterogeneity. Meanwhile, only those with higher depression scores above the fifth quantile statistically significantly benefit from Internet use. It suggests that Internet use promotes mental health more effectively for disadvantaged older people than their counterparts with better health. Specifically, Internet use is associated with an increase in older people’s mental health ranging from 0.678 to 2.220 for the remaining half of older people with poorer mental health, which is significantly larger than the mean effect in OLS.

**TABLE 4:** Quantile regression results for the effect of Internet use on depression in older people (China Health and Retirement Longitudinal Study, China, 2018).

Variable name	Quantiles								
	0.1	0.2	0.3	0.4	0.5	0.6	0.7	0.8	0.9
Internet use	−0.064 (0.908)	−0.137 (0.272)	−0.102 (0.247)	−0.156 (0.297)	−0.385 (0.235)	−0.678*** (0.251)	−1.134** (0.464)	−1.678** (0.659)	−2.220*** (0.505)
Gender	−0.693*** (0.138)	−0.804*** (0.140)	−1.105*** (0.163)	−1.320*** (0.187)	−1.700*** (0.196)	−2.116*** (0.220)	−2.328*** (0.258)	−2.257*** (0.290)	−2.434*** (0.323)
Age	0.010 (0.014)	0.007 (0.013)	0.004 (0.014)	0.009 (0.016)	−0.001 (0.018)	−0.020 (0.020)	−0.023 (0.023)	−0.022 (0.025)	−0.031 (0.034)
Ethnicity	0.021 (0.244)	−0.0165 (0.186)	−0.558*** (0.201)	−0.656 (0.465)	−0.428 (0.723)	−0.545* (0.282)	−0.365 (0.591)	−0.369 (2.590)	−0.193 (8.003)
Education attainment (illiterate)									
Primary Schools	−0.220 (0.146)	−0.274* (0.165)	−0.352* (0.200)	−0.257 (0.222)	−0.399 (0.264)	−0.448 (0.278)	−0.576** (0.284)	−1.069*** (0.324)	−1.021** (0.420)
Junior High School	−0.418 (0.304)	−0.633*** (0.241)	−0.764*** (0.264)	−0.940*** (0.300)	−1.286*** (0.338)	−1.510*** (0.379)	−1.964*** (0.396)	−2.516*** (0.421)	−2.999*** (0.501)
High School and above	−0.730 (0.679)	−1.134*** (0.318)	−1.486*** (0.313)	−1.809*** (0.373)	−2.149*** (0.413)	−2.459*** (0.471)	−2.662*** (0.513)	−2.927*** (0.580)	−2.731*** (0.665)
Religiosity	−0.225 (0.163)	−0.414*** (0.157)	−0.584*** (0.184)	−0.754*** (0.235)	−0.892*** (0.223)	−0.984*** (0.222)	−0.911*** (0.278)	−1.117*** (0.385)	−0.893** (0.371)
Residence	−0.646*** (0.239)	−1.017*** (0.165)	−1.385*** (0.170)	−1.461*** (0.207)	−1.455*** (0.255)	−1.428*** (0.275)	−1.280*** (0.353)	−1.013*** (0.392)	−1.034*** (0.356)
Marital status	−0.535*** (0.160)	−0.655*** (0.179)	−0.829*** (0.213)	−0.949*** (0.222)	−1.205*** (0.231)	−1.609*** (0.272)	−1.702*** (0.304)	−1.883*** (0.354)	−2.025*** (0.495)
Residency status	0.016 (0.127)	−0.047 (0.131)	−0.062 (0.146)	−0.036 (0.162)	−0.052 (0.180)	−0.074 (0.206)	−0.143 (0.236)	−0.103 (0.279)	−0.253 (0.307)
Constant term	1.926 (1.595)	4.434***(1.017)	6.995*** (1.040)	−0.754*** (1.253)	11.517*** (1.566)	15.745*** (1.580)	18.851*** (2.009)	22.540*** (3.507)	27.592*** (7.993)
Observations	8,505	8,505	8,505	8,505	8,505	8,505	8,505	8,505	8,505

Note: ***, **, * denote 1%, 5% and 10% significance levels respectively; all coefficients and S.E. are unstandardized.

To detail the heterogeneity of the health promotion effect, we examine whether Internet use’s effect varies by gender and residence areas, as shown in Table 5. For simplicity, the coefficients of all control variables are not reported. The results show that there are considerable heterogeneities between places of residence. Compared to older people in rural areas, those in urban ones benefit from Internet use significantly. Rural older people in each quantile are non-significant at a 10 percent level. A confusing picture of heterogeneity is shown between genders. The coefficients of women are larger than men’s but with significance at a 10 percent level. Thus the unequal heterogeneity of the health promotion effect between genders cannot be confirmed in this study.

**TABLE 5:** Quantile regression results by gender and residence areas (China Health and Retirement Longitudinal Study, China, 2018).

Variable name		Quantiles								
		0.1	0.2	0.3	0.4	0.5	0.6	0.7	0.8	0.9
Internet use	Urban residents	−0.041 (0.371)	−0.067 (0.303)	−0.110 (0.319)	−0.134 (0.309)	−0.263 (0.263)	−0.491 (0.300)	−0.933* (0.486)	−1.466** (0.701)	−1.830** (0.823)
	Rural residents	−0.104 (0.917)	−0.464 (0.460)	−0.500 (0.516)	−0.283 (0.564)	−0.486 (0.540)	−0.885 (0.590)	−1.353 (0.843)	−1.904 (1.461)	−1.842 (3.786)
	Women	−0.172 (1.487)	−0.325 (0.604)	−0.473 (0.565)	−0.825 (0.615)	−0.946** (0.474)	−0.943* (0.494)	−1.327 (0.929)	−2.100** (0.852)	−2.967* (1.716)
	Men	0.008 (0.809)	−0.006 (0.303)	0.024 (0.268)	−0.021 (0.308)	−0.245 (0.273)	−0.576* (0.301)	−0.960** (0.468)	−1.431 (1.069)	−1.954*** (0.522)
Control variables		Yes								
N		8,505	8,505	8,505	8,505	8,505	8,505	8,505	8,505	8,505

Note: ***, **, * denote 1%, 5% and 10% significance levels, respectively.

### Sensitivity Analysis

This study mainly selected older people aged 60–80 as the subject of the analysis, ignoring some potential vital samples. So we perform robustness tests under different sample group restrictions following Rafnsson et al. (34) reported in Table 6. First, we extend the sample to include those aged 80 and over, including 9,070 cases. The results show that the coefficient of Internet use is −0.859 with a statistical significance at a 0.01 percent level, slightly larger than above (−0.854). Coefficients of Internet use in IVQR are negatively correlated with the depression scores, which is also in line with the above. Second, we extend the lower age limit to 50. We obtain a very similar result and more coefficients in lower quantiles are significant due to the larger sample.

**TABLE 6:** Results of the sensitivity analysis (China Health and Retirement Longitudinal Study, China, 2018).

	2SLS	IVQR								
		Quantiles								
		0.1	0.2	0.3	0.4	0.5	0.6	0.7	0.8	0.9
Test I. Sample aged 60 years and above										
Internet use	−0.859*** (0.207)	−0.068 (0.917)	−0.185 (0.265)	−0.198 (0.238)	−.266 (0.290)	−0.459* (0.235)	−0.731*** (0.249)	−1.178*** (0.449)	−1.662*** (0.576)	−2.017*** (0.502)
Observations	9,070	9,070	9,070	9,070	9,070	9,070	9,070	9,070	9,070	9,070
Test II. Sample aged 50–80 years										
Internet use	−0.748** (0.232)	−0.138 (0.381)	−0.286 (0.186)	−0.371** (0.167)	−0.424** (0.199)	−0.554*** (0.199)	−0.692*** (0.190)	−0.963*** (0.304)	−1.300*** (0.368)	−1.487*** (0.455)
Observations	14,021	14,021	14,021	14,021	14,021	14,021	14,021	14,021	14,021	14,021

Note: ***, **, * denote 1%, 5% and 10% significance levels, respectively; all coefficients and S.E. are unstandardized.

## Discussion

Using nationally representative and the latest available data from China in 2018, this study examines the impact of Internet use on older people’s mental health and the impact heterogeneity instead of focusing on average impacts conducted by previous research. It allows us to deeply understand to what extent Internet use unevenly affects the mental health of different older people. We find that less than 10 percent of older adults used the Internet, which, however, generally positively impacts older people’s mental health (35, 36). Increasing the frequency of Internet use can effectively alleviate the mental depression of older people and enhance their mental health. Each increase in the frequency level of Internet use can reduce the depression score of older people by approximately half a point. Depression scores of older people using the Internet were 5.46, significantly lower than 6.68 for Internet non-users. Our findings are consistent with previous studies from both China and other countries. Results from Yang and his colleagues (29) based on the CHALRS indicate that older adults using the Internet had 1.8 points lower scores of depression symptoms than non-users. Cotten et al. (7) estimated a one-third reduction in the depression probability among older cyber users in the United State. In Japan, 11.7 percent of Internet non-users reported clinical depression, whereas 7.7 percent of Internet users developed clinical depression (37).

Those findings align with the activity theory, which claims that older people should take on specific social roles in later life and remain active and fully socially engaged as a key and fundamental part of successful aging (38). Internet use has effectively enriched opportunities for older people to participate socially and reduced social isolation through multiple pathways of social engagement, such as learning, entertainment, social interaction, and access to information (39). It is essential for older people to connect with social networks leading to a reduction in depressive symptoms and an increase in mental health (40). The Internet is a critical medium for contact and interaction with their children, especially for empty-nest older adults with more serious mental health problems (41). In addition, they also use the Internet to gain and learn about health and ways to channel their emotions to promote their mental health (42).

However, there is enormous heterogeneity in the effects on older adults with different mental health conditions. Internet use only has a statistically alleviating effect on older people with poor mental health. Furthermore, the marginal effect decreases with health conditions. The more poorly they did, the stronger the alleviating effect was, while the effect of Internet use on their mental health was not significant for those with better health conditions. The effect of Internet use on older people with better mental health was insignificant, which may be attributed to two reasons. On the one hand, as “ignorance is bliss” says, happy people would self-select into low attachment to Internet use and be content with their current lives without seeking to improve their situation through the Internet (43). On the other hand, The Internet hardly impacts older people’s mental health, such as emotional isolation, if they only rely on weak online relationships. The health promotion effect occurs when weak online relationships are transformed into more solid offline relationships, which are more likely to provide emotional support and thus alleviate mental health problems (18). While the ability of the Internet to transform online relationships into offline relationships is weakening, resulting in a reduction in meeting emotional needs. For older users with better health conditions, Internet use weightily affects the “information flow” rather than the “emotional flow” (19). They may be more active in offline social engagement activities while focusing on the information itself through the Internet (44). Internet, when the positive effects of offline activities on their mental health outweigh the ones from the Internet, can lead to a situation where the effect on users’ mental health is feeble or disappears.

We also found unequal heterogeneities between places of residence and genders. Rural older adults did not seem to alleviate depression symptoms by using the Internet, which can be attributed to several factors. First, the non-significance for rural older people may be rooted in the small sample size. As small as 2.7 percent of older people in rural areas used the Internet, compared to 21.5 percent in urban ones. Second, it may be related to the limited access to the Internet for rural older adults in the real world. Most rural older adults do not use smartphones or computers. Third, the minority of older people with Internet access exclusively use a small number of simple functions such as WeChat.

### Conclusion

This study confirmed the positive and heterogeneous impact of Internet use on older people’s mental health by using a highly representative and latest available dataset. Specifically, older adults with poorer health conditions are more beneficial from the Internet. Furthermore, compared to older adults in urban areas, those in rural ones did not show the buffering effect of Internet use. Our findings shed light on active and healthy aging strategies. Firstly, the Internet user environment should be improved in parallel with Internet technology. Over 110 million users are older people aged 60 and over by the end of 2020 in China. Due to the strictest quarantine policy in the world, this number is expected to double or triple in recent years. While the Chinese government at different levels has exerted much effort to let older adults gain access to the Internet, further efforts should be in improving the ability of older adults to use the Internet effectively. The government should make top-level design guidelines with a guarantee of including older adults, especially those in less developed areas such as rural areas, and the disadvantaged group such as older women. For example, we should carry out the age-appropriate renovation of the Internet environment to improve Internet accessibility for older people in rural areas or with less education.

Secondly, Multiple approaches should be taken to alleviate the mental health problems of older people with diverse mental health conditions, and various supports should be provided for their Internet use. For older people with poor mental health, we should narrow the digital divisions from triple aspects. One is improving Internet accessibility to overcome the first digital division by expanding Internet coverage and reducing user costs. Another measure has to aim at the acceptability of the Internet. It includes simplifying digital devices. Despite the rapid development of Internet technology in current years, most digital products and their functions target younger users and neglect the needs of older people. We should accelerate the development of age-appropriate Internet products and services to meet the growing demand for internet access and the emotional needs of older people. The third may lie in improving “digital literacy,” which enables the virtual network to be transformed into offline network efficiency. Inequality in socioeconomic status and knowledge also exists in the virtual community, which produces stratified outcomes for subgroups. Older people with mental health problems can engage in social activities through the Internet, replacing the decreased accessibility of offline social networks.

### Limitations

There are also some limitations in this study. Firstly, given the subject of our research, it is difficult to find appropriate external shocks (e.g., unanticipated policy changes) as Instrumental variables for Internet use. As such, the validity of our Instrumental variable could be questioned, although its validity has been technologically passed in our test. More importantly, given the proximity of the OLS and the 2SLS estimates, we believe that our results are not heavily contaminated by endogeneity, and the positive impact of Internet use on older adults’ mental health can be interpreted as a causal relationship. Secondly, this study only uses the frequency of Internet use in older people to measure the independent variables. It does not consider the varied effects of different Internet use behaviors of older people on mental health since the limitation of current questionnaires. We failed to test the linking mechanism between Internet use and mental health. Thirdly, due to the low usage rate of the Internet in earlier years, less than 10 percent of older people have access to the Internet, so the generalizability of our findings is likely to be questioned. However, existing studies based on several national surveys, including CLASS, CFPS, and CGSS, have together confirmed the positive association between Internet use and mental health (30, 45, 46). With the universalization of the Internet, those limitations deserve to be resolved in further study.

## References

[1] National Bureau of Statistics. The 7^th^ Population Census Report (2021). Available from: http://www.stats.gov.cn/tjsj/pcsj/rkpc/d7c/202111/P020211126523667366751.pdf (Accessed December 10 2022).

[2] Sheng Y Gu D. Probabilistic Population Projection and its Application: An Introduction of Methods Used in the UN World Population Prospects. J Popul (2020) 42(5):31–46. 10.16405/j.cnki.1004-129X.2020.05.003

[3] Zhao J Guan W Wang J. The Effect of “Reverse Feeding” on the Mental Health of the Elderly. J Jinan (Philos Soc Sci) (2022) 44(2):39–55. 10.3969/j.issn.1000-5072.2022.02.004

[4] Sun X Sun J Chen L Liu L. Characteristics of Health Needs and Health Management Countermeasures for Older People. Chin J Gerontol (2018) 38(21):5364–7. 10.3969/j.issn.1005-9202.2018.21.091

[5] CNNIC. 49^th^ China Statistical Report on Internet Development (2021). Available from: http://www.cnnic.net.cn/NMediaFile/old_attach/P020220721404263787858.pdf (Accessed December 10 2022)

[6] van Boekel LC Peek ST Luijkx KG. Diversity in Older Adults’ Use of the Internet: Identifying Subgroups through Latent Class Analysis. J Med Internet Res (2017) 19(5):e180. 10.2196/jmir.6853 28539302 PMC5463053

[7] Cotten SR Ford G Ford S Hale TM. Internet Use and Depression Among Retired Older Adults in the United States: A Longitudinal Analysis. J Gerontol B Psychol Sci Soc Sci (2014) 69(5):763–71. 10.1093/geronb/gbu018 24671896

[8] Xu X Li Z Gao Q. Does Internet Use Affect the Loneliness of Older People? an Empirical Study Based on CHARLS Data. J Shandong Univ Financ Econ (2021) 33(3):100–8. 10.3969/j.issn.1008-2670.2021.03.010

[9] Liu J Guo C. The Impact of Mobile Internet Application (APP) Use on the Physical and Mental Health of the Elderly: Focusing on WeChat, WeChat Moments and Mobile Payments. Popul Dev (2021) 27(6):117–28.

[10] Hou J Zhou W. Mechanisms and Heterogeneity Analysis of the Impact of Internet Use on Health Status of the Elderly in China. J Popul (2022) 44(3):73–87. 10.16405/j.cnki.1004-129X.2022.03.006

[11] Kraut R Patterson M Lundmark V Kiesler S Mukophadhyay T Scherlis W. Internet Paradox: A Social Technology that Reduces Social Involvement and Psychological Well-Being? Am Psychol (1998) 53(9):1017–31. 10.1037//0003-066x.53.9.1017 9841579

[12] Dickinson A Gregor P. Computer Use Has No Demonstrated Impact on the Well-Being of Older Adults. Int J Hum Comput Stud (2006) 64(8):744–53. 10.1016/j.ijhcs.2006.03.001

[13] Nie NH. Sociability, Interpersonal Relations, and the Internet: Reconciling Conflicting Findings. Am Behav Sci (2001) 45(3):420–35. 10.1177/00027640121957277

[14] DiNardi M Guldi M Simon D. Body Weight and Internet Access: Evidence from the Rollout of Broadband Providers. J Popul Econ (2019) 32(3):877–913. 10.1007/s00148-018-0709-9

[15] Wan Z He B He X Wang W Zhang H. The Relationship Among Smartphone Use, Phone Dependence and Loneliness of the Elderly in Community Daycare Centers. Mod Prev Med (2021) 48(1):86–9.

[16] Jiang N Xie H Qian R Ye H. Progress in Research on Loneliness Among Urban Older Adults. Chin J Gerontol (2019) 39(4):1006–8. 10.3969/j.issn.1005-9202.2019.04.076

[17] Ma X Chen C. A Survey of the Current Situation of Psychological Frailty and Loneliness in the Elderly. Chin Health Serv Manage (2020) 37(2):138–40.

[18] Xie B. The Mutual Shaping of Online and Offline Social Relationships. Inform Res (2008) 13(3):1–18.

[19] Chen H Chen T. A Comparative Study of the Economic Growth Effects of Outward FDI from BRICS and Developed Countries: An Analysis Based on A Dynamic Panel Instrumental Variable Approach. J Int Trade (2018)(4) 72–89. 10.13510/j.cnki.jit.2018.04.007

[20] Hong J Huang F Pi Z. Internet Use and Psychological Well-Being Among Old Adults. J Huazhong Normal Univ (Humanit Soc Sci Ed) (2015)(2) 171–6.

[21] Ran X Hu H. Urban-Rural Disparity, Digital Divide and Health Inequality of the Elderly. J Popul (2022) 44(3):46–58. 10.16405/j.cnki.1004-129X.2022.03.004

[22] Zhao J Liu Z. The Impact of Internet Use on the Health of Older People. Chin J Popul Sci (2020)(5) 14–26.

[23] Li J Zhou X. Internet Use and Chinese Older Adults’ Subjective Well-Being (SWB): The Role of Parent-Child Contact and Relationship. Comput Hum Behav (2021) 119:106725. 10.1016/j.chb.2021.106725

[24] Li Z Jia C. The Impact of Internet Use on the Mental Health of Middle-Aged and Elderly People: An Examination of Heterogeneous Characteristics and Mechanisms of Action. Jiangsu Soc Sci (2021)(6) 72–9. 10.13858/j.cnki.cn32-1312/c.20211124.022

[25] Guo J Xu Y Chen S Zhu L. Study on the Effect of Internet Use on Loneliness Among the Elderly: The Moderating Role of Age. Chin Health Pol Res (2021) 14(8):29–36. 10.3969/j.issn.1674-2982.2021.08.005

[26] Miller WC Anton HA Townson A. Measurement Properties of the CES-D Scale Among Individuals with Spinal Cord Injury. Spinal Cord (2008) 46(4):287–92. 10.1038/sj.sc.3102127 17909558

[27] Cao P Luo H Hou L Ren X. Depressive Symptoms in the Mid- and Old-Aged People in China. J Sichuan Univ (Med Sci) (2016) 47(5):763–7. 10.13464/j.scuxbyxb.2016.05.027 28598095

[28] Wood AM Joseph S. The Absence of Positive Psychological (Eudemonic) Well-Being as a Risk Factor for Depression: A Ten-Year Cohort Study. J Affect Disord (2010) 122(3):213–7. 10.1016/j.jad.2009.06.032 19706357

[29] Yang H Zhang S Cheng S Li Z Wu Y Zhang S A Study on the Impact of Internet Use on Depression Among Chinese Older People under the Perspective of Social Participation. BMC Geriatr (2022) 22(22):701. 10.1186/s12877-022-03359-y 35999498 PMC9398499

[30] Zhang C Wang Y Wang J Liu X. Does Internet Use Promote Mental Health Among Middle-Aged and Older Adults in China? Front Psy (2022) 13:999498. 10.3389/fpsyg.2022.999498 PMC9706203 36457930

[31] Chen Y. Logic, Imagination and Interpretation: The Application of Instrumental Variables to Causal Inference in the Social Sciences. Sociol Stud (2012)(6) 192–216. 10.19934/j.cnki.shxyj.2012.06.009

[32] Chernozhukov V Hansen C. Instrumental Variable Quantile Regression: A Robust Inference Approach. J Econom (2008) 142(1):379–98. 10.1016/j.jeconom.2007.06.005

[33] Li Y. The Inertia of Medical Care Conducts and the Unfair of the Utilization of the Health Care Services in the Elderly between the City and Countryside. Northwest Popul (2016) 37(2):5–10. 10.15884/j.cnki.issn.1007-0672.2016.02.002

[34] Rafnsson SB Shankar A Steptoe A. Longitudinal Influences of Social Network Characteristics on Subjective Well-Being of Older Adults: Findings from the ELSA Study. J Aging Health (2015) 27(5):919–34. 10.1177/0898264315572111 25804898

[35] Zhou X Wang X. The Influence of Internet Usage on the Life Satisfaction of Rural Elderly: Empirical Analysis Based on CLASS 2016 Data. J Fujian Agri Univ (Philos Soc Sci) (2020) 23(6):70–8.

[36] Lv M Peng X Zhang Y. The Internet and the Health of the Elderly in Rural Areas: Microscopic Evidence and Mechanisms of Influence. Chin Econ Stud (2022)(04) 156–69. 10.19365/j.issn1000-4181.2022.04.12

[37] Nakagomi A Shiba K Kondo K Kawachi I. Can Online Communication Prevent Depression Among Older People? A Longitudinal Analysis. J Appl Gerontol (2022) 41(1):167–75. 10.1177/0733464820982147 33356760

[38] Havighurst RJ. Successful Aging. Process Aging Soc Psychol Perspect (1963) 1:299–320. 10.1093/geront/37.4.433

[39] Shi T Zhang P Han Y. Association between Social Isolation and Depressive Symptoms Among the Elderly People in Community: a Longitudinal Study. Mod Prev Med (2021) 48(17):3154–6.

[40] Ni C Wang Z. The Impact of Internet Use on the Social Isolation in Older Adults. J Popul (2022) 44(3):59–72. 10.16405/j.cnki.1004-129X.2022.03.005

[41] Zhang J AruhanWu X. Relation of Depressive Symptoms to Intergenerational Support and Internet Use in Empty Nest Elderly. Chin J Ment Health (2021) 35(10):838–43. 10.3969/j.issn.1000-6729.2021.10.007

[42] Cohall AT Nye A Moon-Howard J Kukafka R Dye B Vaughan RD Computer Use, Internet Access, and Online Health Searching Among Harlem Adults. Am J Health Promot (2011) 25(5):325–33. 10.4278/ajhp.090325-QUAN-121 21534835

[43] Binder M Coad A. From Average Joe's Happiness to Miserable Jane and Cheerful John: Using Quantile Regressions to Analyze the Full Subjective Well-Being Distribution. J Econ Behav Organ (2011) 79(3):275–90. 10.1016/j.jebo.2011.02.005

[44] Mao X. A Comparative Analysis of Social Participation of Older People in Urban and Rural Areas and its Influencing Factors: Based on CHARLS Data. Rural Econ Sci Technol (2021) 32(7):221–3. 10.3969/j.issn.1007-7103.2021.07.082

[45] Wang L. Internet Use, Leisure Preference and Rural Residents' Happiness. J Harbin Univ Commer (2018)(4) 26–34.

[46] Tang D Zhang K Qi X. The Impacts of Internet Use on Social Networks and Loneliness Among Older Adults: A Study Based on the Purposes of Internet Use. Popul Res (2022)(3) 88–101.

