# Supplementary material for: The Heterogeneous Impact of Internet Use on Older People’s Mental Health: An Instrumental Variable Quantile Regression Analysis
Source: Int J Public Health. 2023 Mar 7;68:1605664. doi: 10.3389/ijph.2023.1605664 (PMC10027613; doi:10.3389/ijph.2023.1605664)
Supplement: Supplementary file 1 [file Table1.DOCX]

**Appendix 1 Sample distribution with missing data (N=671)**

| Variables | Minimum  value | Maximum  value | Average  value | Standard  deviation |
| --- | --- | --- | --- | --- |
| Gender | 0 | 1 | 0.45 | 0.50 |
| Age | 60 | 80 | 70.05 | 6.140 |
| Ethnicity | 0 | 1 | 0.92 | 0.28 |
| Education attainment | 1 | 4 | 1.84 | 0.85 |
| Religiosity | 0 | 1 | 0.18 | 0.39 |
| Marital status | 0 | 1 | 0.77 | 0.43 |
| Coresidence with children | 0 | 1 | 0.60 | 0.49 |
| Residence | 0 | 1 | 0.26 | 0.44 |
